# Primary Care Provider Evaluation of Cancer Survivorship Care Plans Developed for Patients in their Practice

**DOI:** 10.4172/2329-9126.1000163

**Published:** 2014-05-26

**Authors:** Kim L. Dittus, Brian L. Sprague, Claire M. Pace, Dorothy A. Dulko, Lori A. Pollack, Nikki A. Hawkins, Berta M. Geller

**Affiliations:** 1 Hematology/Oncology Division, University of Vermont, College of Medicine, Burlington, USA; 2 Department of Surgery and Office of Health Promotion Research, University of Vermont, Burlington, USA; 3 Department of Radiation Oncology, Dartmouth Medical School, Lebanon, USA; 4 Memorial Sloan Kettering Cancer Center, New York, New York, USA; 5 Centers for Disease Control and Prevention, Division of Cancer Prevention and Control, Atlanta, USA; 6 Family Medicine and Radiology Departments, University of Vermont, College of Medicine, Burlington, USA

**Keywords:** Cancer survivor, Health communication, Care transitions

## Abstract

**Objective:**

Survivorship care plans (SCP), which describe a cancer survivor’s diagnosis, treatment and follow-up, are recommended. The study objective was to evaluate primary care providers’ (PCP) responses to SCPs developed for breast and colorectal cancer survivors in their practice and to determine whether PCP response to the SCPs varied according to characteristics of the practitioner and their practice.

**Method:**

SCPs were created using the Journey Forward® Care Plan for breast and colorectal cancer patients in rural and urban settings. The SCP and a survey were sent to PCPs.

**Participants:**

Primary care physicians.

**Main Measures:**

Attitudes regarding survivorship care plans.

**Results:**

Thirty-nine (70.9% response rate) surveys were completed. Most felt the SCP was useful (90%), that it enhanced understanding (75%) and that detail was sufficient (>80%). However, 15% disagreed that the care plan helped them understand their role, a perception especially prevalent among PCPs in the rural setting. Among PCPs with ≤ 18 years in practice, 95% agreed that the SCP would improve communication with patients, contrasted with 60% of those with >21 years in practice. The most common barrier to providing follow-up care was limited access to survivors.

**Conclusions:**

While SCPs appear to improve PCPs understanding of a cancer diagnosis and treatment, clear delineation of each provider’s role in follow-up care is needed. Additional detail on which tests are needed and education on late and long term effects of cancer may improve coordination of care.

## Introduction

Improvements in cancer screening and therapy have resulted in a growing number of cancer survivors. The transition between initial therapy and surveillance has unique challenges [1]. Oncology visits often continue but focus on cancer issues and do not include screening for other malignancies or general health monitoring. Previous research suggests that when care is shared between specialists and primary care providers (PCPs), survivors receive better health care [2–4]. Yet, the role of the PCP in survivorship care is often unclear and communication is inadequate between the oncologist, PCP and patient [5–7].

The 2005 Institute of Medicine report on cancer survivorship, From Cancer Patient to Cancer Survivor: Lost in Transition called for the development of cancer survivor documents for survivors and their PCPs [8]. In 2015 the American College of Surgeons Commission on Cancer will require a survivorship care plan (SCP) for cancer survivors [9]. A SCP has the potential to facilitate communication about the oncology experience. Ideally, it provides a clear outline of the diagnosis, treatments received, plan for monitoring and clarifies the roles of the medical providers involved in the survivor’s care.

PCPs clearly favor the receipt of a summary of cancer diagnosis and care [6,10] and their perceptions of SCPs have been positive [11]. However, there is limited research evaluating how PCPs perceive SCPs developed for their own patients. It is also unknown whether the needs and preferences of PCPs in different practice settings differ regarding follow up care of cancer survivors. We evaluated PCPs’ understanding of and satisfaction with SCPs prepared for breast and colorectal cancer survivors in their practice. Additionally we examined whether PCP response to the SCPs varied according to characteristics of the practitioner and their practice.

## Methods

SCPs were developed for breast and colorectal cancer patients and mailed to their PCPs at an urban and rural cancer center. In 2010, the urban area had a census of greater than 150,000, while the rural area had a population of approximately 31,000. All stage 0–3 breast cancer and stage 2–4 colorectal cancer patients who received intent to cure oncologic interventions between January 2011 and July 2011 were eligible to receive a SCP. A total of 89 patients were invited to participate in the study and 78 agreed (88%), including 61 with breast cancer and 17 with colorectal cancer.

### Document development and delivery

Journey Forward^®^ software was used to create the SCP [12]. The content followed the IOM report recommendations to include a summary of cancer type, treatment and treatment related complications, recommendations for follow up and the type of provider to see for follow up care, information on secondary cancer prevention and health promotion, and local resources [8]. Diagnosis and treatment information was obtained from medical records. The document was provided to both the cancer survivor and their PCP.

Prior to study initiation two survivor focus groups (one for breast cancer participants, one for colorectal cancer participants) were conducted to review sample Journey Forward care plans and the explanatory material for SCPs. Focus group participants were recruited from the Vermont Cancer Survivor Registry [13]. All focus groups lasted approximately 90 minutes. Additionally, three PCPs reviewed SCP templates and the survey for ease of use and completeness.

The SCP documents were prepared at each cancer center by an advanced practice provider (Nurse Practitioner or Physician Assistant) who had training in cancer survivor issues. The same practitioner also presented and discussed the SCP with cancer survivors. If inconsistencies in the SCP were identified by the patient at the time of the visit the information in question was re-examined and corrected in the SCP.

### Survivor and PCP recruitment

At the urban center all eligible patients presenting for a first or second post therapy visit were informed that their next clinic visit would be an hour long survivorship visit which would include usual review of side effects and monitoring and a discussion of the SCP. At the visit they were informed of the opportunity to participate in a study to evaluate the SCP. If they agreed to participate in the longer visit and receive the document, an informed consent was signed. At the rural center patients were approached at the end of initial oncologic therapy and asked if they would like to receive a separate survivor visit and SCP. If the patient signed consent they were scheduled for a 1 hour long survivor visit 1–3 months later (i.e., same time frame as for the urban clinic visit). Several days prior to the visit, patients at both sites received a reminder call that the appointment was approaching.

### PCP Survey development and administration

A survey was developed by study investigators to evaluate PCPs perceptions of the SCPs they received for patients in their care. The survey included the following topics: understanding of the patient’s treatment and follow up; adequacy of content detail; communication; and barriers to caring for cancer survivors. The survey used a 5-point Likert scale with five responses ranging from “Strongly Agree” to “Strongly Disagree”. Participants were asked to circle the number corresponding to their level of agreement with each statement. Demographic information about their practice was collected.

The SCP was mailed to the PCPs with a cover letter explaining the study, the survey and a self-addressed stamped return envelope. The return of the survey implied consent at the urban center. At the rural center PCP’s returning the survey also returned a signed IRB approved consent. If a PCP received two SCPs he/she only received a survey with the first SCP. If the survey was not returned, two attempts were made to contact the PCP by mail. PCPs who did not return the survey within 4–8 weeks were contacted via email by an oncologist involved in the study at the urban center, while at the rural center an administrative secretary called the PCP.

### Statistical analysis

Descriptive statistics were calculated to determine survey response frequencies and the distribution of demographic variables. Likert scale responses for strongly agree and agree as well as strongly disagree and disagree were collapsed in order to obtain categories with sufficient sample sizes for analysis. Descriptive statistics were used to assess differences between urban and rural PCPs responses and between those in practice less than or equal to and greater than the median of 18 years. Fisher’s exact test was used to identify statistically significant (p<0.05) differences according to these factors. All statistical analyses were conducted using SAS Statistical Software (Version 9; SAS Institute, Inc., Cary, North Carolina).

## Results

Of the 55 surveys sent to PCPs, 39 were returned for a response rate of 70.9%. We received 22 surveys from PCP’s in the urban area and 17 from PCP’s in the rural area. Demographic characteristics of respondents are summarized in Table 1. The majority of respondents were female and family practice physicians. The demographic characteristics between rural and urban centers were similar other than a predominance of female providers in the rural area.

Nearly all PCP's reported that the SCP was easy to understand (97%), incorporated appropriate topics (97%) and was useful (90%). However 16% disagreed that the length of the SCP (which was on average 5 pages) was "just right". Written comments concurred that several PCPs felt the SCP was too long.

### Understanding SCP information

Greater than 75% of PCP’s felt the SCP contributed to their understanding of the cancer diagnosis, treating team, recommendations for follow up, and resources available for survivorship care, with no statistically significant differences between urban and rural sites (Table 2).

PCPs in practice >18 years were significantly less likely to endorse that the SCP contributed to their understanding of the cancer diagnosis (p-interaction=0.04) and were less likely to agree that they understood the follow-up for late or chronic problems resulting from cancer treatment (p-interaction=0.02). A proportion of respondents (15%) disagreed that the care plan helped them understand their role in facilitating survivorship care and 15% disagreed that they understood how responsibility would be shared with the oncologist. The PCPs from the rural site were less likely to agree that the SCP contributed to their understanding of their role (p-interaction=0.05).

### Adequacy of content detail

The survey assessed whether the level of detail in the SCP was adequate to perform several tasks (Table 3). Greater than 80% of respondents agreed that the level of detail was sufficient and response was similar for years in practice. However, the rural PCP’s were less likely than the urban PCPs to agree that the SCPs provided sufficient detail about monitoring for late and long term effects and tests to obtain for monitoring. There was no significant difference in the perception of SCP content detail by years in practice.

### Communication

Over 70% of PCP’s at each site agreed that SCPs would improve communication with medical providers and patients (Table 4).

Perceptions about improved communication with patients were similar among urban and rural PCPs, but differed according to years in practice. Among those with ≤ 18 years in practice, 95% of PCPs agreed that the SCP would improve communication with patients, whereas only 60% of PCPs with >18 years in practice agreed (p-interaction= 0.03). Despite general agreement that the SCP was useful, 13% of all respondents either did not care if they received SCP documents or did not want to receive them. There was no statistically significant difference in desire to receive SCPs by site or years in practice.

Barriers to providing survivorship care identified by PCP's are included in Table 5. The most common barrier to providing follow-up care was “limited access to survivors since they stay with their oncologist” with 64% considering this a significant or moderate barrier. PCP’s practicing for less than 18 years were significantly more likely to feel that insufficient knowledge of cancer survivorship issues was a barrier to survivor care (79% vs. 36.8%). Overall, only 18% felt that poor reimbursement was a barrier (Table 5).

## Discussion

In general, PCP’s felt that SCPs developed for their patients contributed to their understanding of the cancer diagnosis and treatment and would improve communication with cancer treatment providers and survivors. However, we discovered differences between urban and rural PCPs and those in practice less than 18 years compared with those in practice longer. We also found that PCPs encounter substantial barriers in providing survivorship care.

Our survey results are consistent with a recent study based on semi-structured interviews of primary care providers receiving SCPs for their patients who felt SCPs increased their knowledge about their patient’s cancer history and recommendations for surveillance [11]. There were two notable exceptions regarding the agreement between the two studies: 1) the role of PCP and oncologist in survivorship care and; 2) division of responsibility for monitoring for recurrence and new primary cancers.

Despite receiving a SCP for a specific patient, many urban and rural PCPs remained unclear about their role in survivorship care and how primary care and oncology might share responsibility. The rural PCPs were significantly less likely to understand their role in follow up care and continuing education efforts should target them. There is not a defined time to transfer the care of a cancer survivor from oncology to primary care. Commonly patients continue to see both a PCP and an oncologist for an extended period making it difficult to clearly delineate who is responsible for follow up care. As a result, many aspects of survivorship follow up may remain ambiguous. Additionally, a majority of PCPs feel that primary care guidelines for adult cancer survivors are not well defined [14].

Significant differences have been reported between oncologists and PCPs in the attitudes, knowledge and practices for cancer survivor care [15]. PCPs express concern that they do not know what surveillance tests are needed or the appropriate duration of surveillance [5,11]. They are also more likely to endorse the use of non-recommended tests for follow up than are oncologists [15]. The SCP should clearly specify which physician is responsible for ordering which monitoring test. This will require clear guidelines from national professional organizations which incorporate input from oncologists and PCPs and define follow up monitoring and the role of the PCP in cancer survivor care. Oncology organizations should be encouraged to establishing clear guidelines delineating responsibility of cancer survivor care.

PCPs in practice a longer time expressed less understanding of the requirements for monitoring for late or chronic problems that result from cancer therapy. As cancer therapy has become more complex, primary care providers further from initial training may be unfamiliar with newer interventions and less aware of the current consequences of cancer therapy. Knowledge of the late and long term impact of cancer therapy is important for PCPs as patients may see them first about these symptoms and late and lingering effects may impact organ systems for which the patient is being monitored.

PCPs generally agreed that the detail in the SCP was adequate. However, PCP’s from the rural area were significantly less likely to agree that the SCP provided sufficient content to help them obtain tests for monitoring. Perhaps PCP’s in the urban area, who were often associated with an academic center, receive more cancer related education or presumed they had easier access to oncologists should questions arise. Additionally, actual monitoring recommendations may be less clearly defined than PCP’s anticipate and recommendations vary by cancer type which contributes to confusion about surveillance.

Insufficient knowledge of cancer survivor issues was a commonly identified barrier among PCPs and may partially explain why they felt the SCP provided insufficient detail. PCP uncertainty about late effects of cancer and follow up care recommendations has been identified by others [15]. Given the uncertainty PCPs have about late effects and what tests are needed for monitoring, continuing medical education should include more information about cancer therapy, long term and late effects of therapy, and surveillance of recurrence and new primary cancers.

Our findings have several limitations. The small sample size limits statistical power to detect small differences in responses by PCP characteristics. The use of a quantitative survey is also a limitation, as a qualitative approach may have provided a richer understanding of the complexities faced by PCPs when using information contained in a SCPs in a busy practice. While the response rate was high, non-respondents may have differed in their assessment of the SCPs. Despite including PCPs practicing in both urban and rural areas, the survey was administered in a geographically localized area which limits generalizability of the study results. Participating PCPs in the urban area may be more integrated with the academic cancer center where most of their patients would receive oncologic care. They may also have more education opportunities and greater access to oncologists thus alleviating concerns about monitoring.

SCPs have the potential to strengthen communication, facilitate care coordination, clarify roles, and lessen PCPs anxiety about providing adequate care, thus increasing the opportunity for more primary care of cancer survivors. PCPs in rural and urban areas appear to find SCPs developed for their patients understandable and the content sufficiently detailed for most topics. However, usefulness of SCPs can be enhanced. Given the time challenges PCPs face SCP should as short and precise as possible. Clear delineation of roles between PCPs and oncologists and specific information within the SCP regarding late and long term effects and monitoring tests are needed. Oncology organizations should be encouraged to develop well-defined follow up recommendations. Ideally a SCP could be updated as new information regarding impacts of cancer treatment and follow up recommendations evolve. A disease and care management model that empowers patients, which improves health care team collaboration and patient outcomes has been found to be beneficial for patient with chronic disease [16] and would likely enhance cancer survivor care. This approach would be a particular advantage in rural settings by helping facilitate appropriate testing and communication with specialists. At a minimum, continuing education regarding cancer survivor issues will help relieve the dissonance PCPs may feel about assuming a larger role in cancer survivor care and their perception of insufficient knowledge of survivor issues and follow up.

## Figures and Tables

**Table 1:** Demographic data on responding primary care providers by practice setting.

Characteristic	Urban (%) n=22	Rural (%) n=17
**Specialty**		
Family Medicine	15 (68.2%)	13 (76.5%)
Internal Medicine	6 (27.3%)	2 (17.7%)
OB/Gyn	1 (4.6%)	0
NP/PA	0	1 (5.9%)
**Gender**		
Male	11 (50%)	5 (29.4%)
Female	11 (50%)	12 (70.6%)
**Median Years of Practice**	17 (range 1–36)	20 (range 10–38)

**Table 2:** Primary care providers’ understanding of components of cancer survivors’ follow up care.

Did the information in the SCP contribute to your understanding regarding:	Total (%) n=39	Urban (%) n=22	Rural (%) n=17	P value	≤ 18 yrs (%) n=19	>18 yrs (%) n=20	P value
**Your patient’s cancer diagnosis**							
Agree	29 (87.9)	18 (81.8)	11 (100)	0.38	17 (100)	12 (75.0)	0.04†
Neutral	2 (6.1)	2 (9.1)	0		0	2 (12.5)	
Disagree	2 (6.1)	2 (9.1)	0		0	2 (12.5)	
**The team who treated your patient for cancer**							
Agree	35 (89.7)	20 (91.0)	15 (88.2)	0.76	19 (100)	16 (80.0)	0.16
Neutral	3 (7.7)	1 (4.5)	2 (11.8)		0	3 (15.0)	
Disagree	1 (2.6)	1 (4.5)	0		0	1 (5.0)	
**The need for routine cancer screening**							
Agree	30 (76.9)	17 (77.3)	13 (76.5)	0.50	17 (89.5)	13 (65.0)	0.15
Neutral	7 (17.9)	3 (13.6)	4 (23.5)		2 10.5)	5 (25.0)	
Disagree	2 (5.1)	2 (9.1)	0		0	2 (10.0)	
**The need for follow-up to assess for late and/or chronic problems resulting from cancer treatment**							
Agree	33 (84.6)	19 (86.4)	14 (82.5)	0.82	19 (100)	14 (70.0)	0.02†
Neutral	3 (7.7)	1 (4.5)	2 (11.8)		0	3 (15.0)	
Disagree	3 (7.7)	2 (9.1)	1 (5.9)		0	3 (15.0)	
**Which follow-up tests will be needed**							
Agree	33 (84.6)	20 (91.0)	13 (76.5)	0.43	18 (94.7)	15 (75.0)	0.21
Neutral	4 (10.3)	1 (4.5)	3 (17.6)		1 (5.3)	3 (15.0)	
Disagree	2 (5.1)	1 (4.5)	1 (5.9)		0	2 (10.0)	
**How often follow-up tests should be performed**							
Agree	35 (89.7)	20 (91.0)	15 (88.2)	0.76	18 (94.7)	17 (85.0)	0.99
Neutral	3 (7.7)	1 (4.5)	2 (11.8)		1 (5.3)	2 (10.0)	
Disagree	1 (2.6)	1 (4.5)	0		0	1 (5.0)	
**The importance of resuming/initiating age appropriate health maintenance practices**							
Agree	31 (79.5)	18 (81.8)	13 (76.5)	0.82	18 (94.7)	13 (65.0)	0.06
Neutral	7 (17.9)	3 (13.6)	4 (23.5)		1 (5.3)	6 (30.0)	
Disagree	1 (2.6)	1 (4.6)	0		0	1 (5.0)	
**Your role in facilitating survivorship care**							
Agree	24 (61.5)	15 (68.2)*	9 (52.9)*	0.05	12 (63.1)	12 (60.0)	0.99
Neutral	9 (23.1)	2 (9.1)	7 (41.2)		4 (21.1)	5 (25.0)	
Disagree	6 (15.4)	5 (22.7)	1 (5.9)		3 (15.8)	3 (15.0)	
**How the PCP and oncology providers will share responsibility for survivorship care**							
Agree	26 (66.7)	15 (68.2)	11 (64.7)	0.79	12 (63.1)	14 (70.0)	0.72
Neutral	7 (17.9)	3 (13.6)	4 (23.5)		3 (15.8)	4 (20.0)	
Disagree	6 (15.4)	4 (18.2)	2 (11.8)		4 (21.1)	2 (10.0)	
**The resources available to cancer survivors/families**							
Agree	30 (78.9)	18 (81.8)	12 (75.0)	0.70	15 (78.9)	15 (78.9)	0.99
Neutral	8 (21.1)	4 (18.2)	4 (25.0)		4 (21.1)	4 (21.1)	
Disagree	0	0	0		0	0	

* Significance between PCP’s from urban and rural practices (p<0.05)

† Significance between PCP’s based on years in practice (p<0.05)

**Table 3:** Primary care providers assessment of the adequacy of detail in survivorship care plans.

Is the level of detail for each area in the SCP sufficient to contribute to your care for cancer survivors?	Total (%) n=39	Urban (%) n=22	Rural (%) n=17	P value	≤ 18 yrs (%) n=19	>18 yrs (%) n=20	P Value
**Cancer treatment care team**							
Agree	37 (94.9)	20 (91.0)	17 (100)	0.99	18 (94.7)	19 (95.0)	0.99
Neutral	1 (2.6)	1 (4.5)	0		0	1 (5.0)	
Disagree	1 (2.6)	1 (4.5)	0		1 (5.3)	0	
**Information about patient risk factors**							
Agree	33 (84.6)	19 (86.4)	14 (82.4)	0.80	17 (89.5)	16 (80.0)	0.99
Neutral	5 (12.8)	2 (9.1)	3 (17.6)		2 (10.5)	3 (15.0)	
Disagree	1 (2.6)	1 (4.5)	0		0	1 (5.0)	
**Cancer specific diagnosis**							
Agree	36 (92.3)	22 (100)	14 (82.4)	0.07	19 (100)	17 (85.0)	0.23
Neutral	3 (7.7)	0	3 (17.7)		0	3 (15.0)	
Disagree	0	0	0		0	0	
**Treatment plan and summary**							
Agree	37 (94.9)	21 (95.5)	16 (94.1)	0.69	18 (94.7)	19 (95.0)	0.99
Neutral	1 (2.6)	0	1 (5.9)		1 (5.3)	0	
Disagree	1 (2.6)	1 (4.5)	0		0	1 (5.0)	
**Follow-up care**							
Agree	36 (94.7)	20 (91.0)	16 (100)	0.50	17 (94.4)	19 (95.0)	0.99
Neutral	0	0	0		0 (5.6)	0	
Disagree	2 (5.3)	2 (9.0)	0		1	1 (5.0)	
**Monitoring for late and long term effects**							
Agree	35 (89.7)	22 (100)	13 (76.5)	0.03*	18 (94.7)	17 (85.0)	0.61
Neutral	4 (10.3)	0	4 (23.5)		1 5.3)	3 (15.0)	
Disagree	0	0	0		0	0	
**Tests to obtain for monitoring**							
Agree	26 (86.7)	21 (95.5)	5 (62.5)	0.01*	14 (93.3)	12 (60.0)	0.60
Neutral	3 (10.0)	0	3 (37.5)		1 (6.7)	2 (13.3)	
Disagree	1 (3.3)	1 (4.5)	0		0	1 (6.7)	
**Symptoms to watch for recurrence or second primary**							
Agree	30 (93.8)	19 (95.5)	11 (91.7)	0.99	15 (93.7	15 (93.7)	0.99
Neutral	0	0	0		0	0	
Disagree	2 (6.3)	1 (5.0)	1 (9.3)		1 (6.3)	1 (6.3)	
**National and community resources**							
Agree	33 (90.9)	20 (91.0)	10 (90.1)	0.99	15 (83.3)	15 (100)	0.23
Neutral	3 (9.1)	2 (9.0)	1 (9.9)		3 (17.7)	0	
Disagree	0	0	0		0	0	

* Significance between PCP’s from urban and rural practices (p<0.05).

**Table 4:** Primary care provider’s assessment of survivorship care plans impact on communication.

	Total (%) N=39	Urban (%) n=22	Rural (%) n=17	P value	≤ 18 yrs (%) n=19	>18 yrs (%) n=20	P value
**Do you think that the SCP will improve your communication with cancer treatment providers?**							
Agree	29 (74.4)	15 (68.2)	14 (82.3)	0.65	14 (73.7)	15 (75.0)	0.99
Neutral	6 (15.4)	4 (18.2)	2 (11.8)		3 (15.8)	3 (15.0)	
Disagree	4 (10.3)	3 (13.6)	1 (5.9)		2 (10.5)	2 (10.0)	
**Do you think that the SCP will improve your communications with cancer survivors?**							
Agree	30 (76.9)	18 (81.8)	12 (70.6)	0.63	18 (94.7)	12 (60.0)	0.03†
Neutral	6 (15.4)	3 (13.6)	3 (17.6)		1 (5.3)	5 (25.0)	
Disagree	3 (7.7)	1 (4.6)	2 (11.8)		0	3 (15.0)	
**Would you like to continue to receive SCPs for your patients?**							
Agree	33 (86.8)	19 (90.5)	14 (82.3)	0.78	18 (94.7)	15 (79.0)	0.41
Neutral	3 (7.9)	1 (5.3)	2 (11.8)		1 (5.3)	2 (10.5)	
Disagree	2 (5.3)	1 (5.3)	1 (5.9)		0	2 (10.5)	

† Significance between PCP’s based on years in practice (p<0.05).

**Table 5:** Barriers primary care providers identify for providing follow up care for cancer survivors.

Barrier that may interfere with PCPs ability to provide follow- up care for cancer survivors	Total (%) n=38	Urban %) n=22	Rural (%) n=17	P value	≤18 yrs (%) n=19	>18 yrs (%) n=20	P value
**Lack of time**							
Significant/Mod. Barrier	17 (44.7)	12 (54.5)	5 (31.3)	0.32	9 (47.4)	8 (42.2)	0.99
Neutral	7 (18.4)	4 (18.2)	3 (18.8)		3 (15.8)	4 (21.1)	
Hardly/Not a Barrier	14 (36.8)	6 (27.3)	8 (50.0)		7 (36.8)	7 (36.8)	
**Insufficient knowledge of cancer survivor issues**							
Significant/Mod. Barrier	22 (57.9)	13 (59.1)	9 (56.3)	0.36	15 (78.9)	7 (36.8)	0.02†
Neutral	8 (21.1)	6 (27.3)	2 (12.5)		3 (15.8)	5 (26.3)	
Hardly/Not a Barrier	8 (21.1)	3 (13.6)	5 (31.3)		1 (5.3)	7 (36.8)	
**Inadequate recommendations from oncology**							
Significant/Mod. Barrier	18 (48.7)	10 (47.6)	8 (50.0)	0.83	11 (61.1)	7 (36.8)	0.36
Neutral	9 (24.3)	6 (28.6)	3 (18.8)		3 (16.7)	6 (31.6)	
Hardly/Not a Barrier	10 (27.0)	5 (23.8)	5 (31.3)		4 (22.2)	6 (31.6)	
**Poor reimbursement for services**							
Significant/Mod. Barrier	7 (18.4)	6 (27.3)	1 (6.3)	0.27	4 (21.1)	3 (15.8)	0.27
Neutral	16 (42.1)	9 (40.9)	7 (43.8)		10 (52.6)	6 (31.6)	
Hardly/Not a Barrier	15 (39.5)	7 (31.8)	8 (50.0)		5 (26.3)	10 (52.6)	
**Limited access to cancer survivors since they stay with oncology practices**							
Significant/Mod. Barrier	25 (64.1)	12 (54.6)	13 (76.5)	0.36	13 (68.4)	12 (60.0)	0.46
Neutral	9 (23.1)	7 (31.8)	2 (11.8)		5 (26.3)	4 (20.0)	
Hardly/Not a Barrier	5 (12.8)	3 (13.6)	2 (11.8)		1 (5.3)	4 (20.0)	
**Survivor care guidelines have not been established**							
Significant/Mod. Barrier	16 (47.1)	9 (42.9)	7 (53.8)	0.87	9 (50.0)	7 (43.8)	0.89
Neutral	15 (44.1)	10 (47.6)	5 (38.5)		8 (44.4)	7 (43.8)	
Hardly/Not a Barrier	3 (8.8)	2 (9.5)	1 (7.7)		1 (5.6)	2 (12.5)	

† Significance between PCP’s based on years in practice (p<0.05).
